# Electrospun Chitosan-Coated Recycled PET Scaffolds for Biomedical Applications: Short-Term Antimicrobial Efficacy and In Vivo Evaluation

**DOI:** 10.3390/polym17081077

**Published:** 2025-04-16

**Authors:** Andreea Mihaela Grămadă (Pintilie), Adelina-Gabriela Niculescu, Alexandra Cătălina Bîrcă, Alina Maria Holban, Alina Ciceu, Cornel Balta, Hildegard Herman, Anca Hermenean, Simona Ardelean, Alexandra-Elena Stoica, Alexandru Mihai Grumezescu, Adina Alberts

**Affiliations:** 1 Department of Science and Engineering of Oxide Materials and Nanomaterials, National University of Science and Technology Politehnica Bucharest, 011061 Bucharest, Romania; g_andreea93@yahoo.com (A.M.G.); adelina.niculescu@upb.ro (A.-G.N.); ada_birca@yahoo.com (A.C.B.); oprea.elena19@gmail.com (A.-E.S.); agrumezescu@upb.ro (A.M.G.); 2 Research Institute of the University of Bucharest—ICUB, University of Bucharest, 050657 Bucharest, Romania; alina.m.holban@bio.unibuc.ro; 3 Faculty of Biology, University of Bucharest, 030018 Bucharest, Romania; 4 “Aurel Ardelean” Institute of Life Sciences, Vasile Goldis Western University of Arad, 310025 Arad, Romania; ciceu.alina@uvvg.ro (A.C.); balta.cornel@uvvg.ro (C.B.); herman.hildegard@uvvg.ro (H.H.); 5 Faculty of Medicine, Vasile Goldis Western University of Arad, 310025 Arad, Romania; 6 Faculty of Pharmacy, Vasile Goldis Western University of Arad, 310025 Arad, Romania; 7 Carol Davila University of Medicine and Pharmacy, 050474 Bucharest, Romania; adina-magdalena.alberts@rez.umfcd.ro

**Keywords:** electrospinning, rPET, chitosan, antimicrobial biomaterials, biofilm inhibition, scaffold, *Staphylococcus aureus*, *Pseudomonas aeruginosa*, *Candida albicans*, tissue integration

## Abstract

This study investigates the preparation of electrospun recycled polyethylene terephthalate (rPET) coated with chitosan (CS) and evaluates their antibiofilm properties and in vivo response. rPET scaffolds were first fabricated via electrospinning at different flow rates (10, 7.5, 5 and 2.5 mL/h) and subsequently coated with chitosan. Scanning electron microscopy (SEM) revealed that fiber morphology varied with electrospinning parameters, influencing microbial adhesion. Antimicrobial tests demonstrated that rPET@CS significantly inhibited *Staphylococcus aureus*, *Pseudomonas aeruginosa* and *Candida albicans* biofilm formation compared to control and uncoated rPET surfaces. Subcutaneous implantation of rPET@CS scaffolds induced a transient inflammatory response, with macrophage recruitment and collagen deposition supporting tissue integration. These findings highlight the potential of rPET@CS scaffolds as sustainable antimicrobial biomaterials for applications in infection-resistant coatings and biomedical implants.

## 1. Introduction

Over the past 7 decades, plastics production has grown at a faster rate than any other industrial material. However, plastics’ durability, adaptability and resistance to degradation make them difficult for nature to break down, leading to significant waste accumulation and severe threats to biodiversity and ecosystems. Specifically, with the rising use of plastic materials, global plastic waste output has increased to 300 million tons per year, with forecasts showing that the amount could quadruple by 2050 [1,2].

Among the wide range of utilized plastic materials, polyethylene terephthalate (PET) is one of the most frequently chosen for a broad range of applications (e.g., beverage and food containers, synthetic fibers for clothing and carpeting, filtering membranes, wound dressings, biosensors, sutures and surgical meshes). Thus, PET-based materials are also among the leading causes of plastic pollution, mainly because of their widespread use in packaging and the relatively short lifecycle of these objects. Thus, special consideration has to be directed to recycling PET and reusing it to generate new end-products [3,4,5,6,7].

Recycled PET is a valuable source material for developing different packaging solutions, textiles and construction materials, and it is a cheap and convenient alternative from the point of view of its properties [8,9,10]. Its physicochemical characteristics are suitable for numerous utilizations, yet some applications may require enhanced features. For instance, using recycled PET for biomedical purposes poses questions about the biocompatibility of the material and the risk of microorganism contamination [11,12,13]. Thus, it is advisable to mix recycled PET with other materials capable of improving its biological behavior for these uses.

Biodegradable polymers such as polylactic acid (PLA), polycaprolactone (PCL) and polyethylene glycol (PEG) have been widely employed in scaffold fabrication due to their tunable mechanical properties, biodegradation rates and compatibility with various fabrication techniques, including electrospinning [14,15,16,17,18,19]. However, these materials are inherently bioinert and lack antimicrobial activity, often necessitating the incorporation of secondary agents such as antibiotics or metallic nanoparticles to achieve infection-resistant properties [20,21,22,23]. Moreover, studies have shown that PEG’s hydrophilic nature can reduce protein adsorption and cell adhesion, which may limit its integration in certain tissue engineering applications [24]. PLA and PCL, while offering mechanical robustness, degrade slowly and may release acidic byproducts that influence the local cellular environment [25,26].

Chitosan, by contrast, has been extensively studied for its intrinsic antimicrobial activity, mucoadhesiveness and ability to promote wound healing. Its polycationic structure enables electrostatic interactions with microbial cell membranes, leading to cell lysis, as supported by both in vitro and in vivo studies [27,28,29]. Recent research has demonstrated that chitosan-based composites can modulate inflammatory responses and accelerate tissue regeneration, especially when combined with structurally supportive polymers to address their limited mechanical strength [30]. These properties make chitosan particularly valuable in the design of multifunctional scaffolds aimed at infection control and tissue repair in biomedical applications [31,32,33,34,35,36].

Electrospinning has emerged as a highly effective technique for producing nanofibrous scaffolds with properties closely mimicking the extracellular matrix (ECM), such as high surface-area-to-volume ratio, tunable porosity and mechanical flexibility [37,38,39]. These features make electrospun membranes particularly suitable for tissue engineering, drug delivery and wound healing applications. Moreover, electrospinning enables the incorporation of bioactive agents into the fibers, which can further enhance biological performance [40]. Recent studies have extensively documented both the advantages and limitations of electrospun nanofiber scaffolds, reinforcing their potential for continued development in biomedical applications [41,42].

In this context, this study aims to merge the benefits of recycled PET and chitosan into developing new biomaterials with medical applicability. The combination of PET and chitosan has been previously reported in the literature, with PET fibers providing the necessary support for chitosan coatings and generating flexible composite fibers with antimicrobial activity [35,43,44,45]. Based on these encouraging findings, our work has addressed the dual challenges of plastic waste management and the need for biocompatible, antimicrobial materials. Specifically, recycled PET and chitosan were combined into electrospun nanofibrous membranes, which were further characterized from physicochemical and biological perspectives.

## 2. Materials and Methods

### 2.1. Materials

The materials used in this study were sourced from Sigma-Aldrich (Darmstadt, Germany) and utilized without further purification. These materials included methylene chloride, trifluoroacetic acid and chitosan (medium molecular weight, 75–85% degree of deacetylation). Polyethylene terephthalate was procured from a used bottle sourced from a local branch of an international beverage brand. The material was thoroughly cleaned and processed to ensure suitability for the intended applications.

### 2.2. Preparation of Electrospun Recycled PET@CS Samples

PET samples were prepared according to our previous published paper [46].

The PET samples were supplementarily covered through the deposition of an additional layer of chitosan. The new samples based on recycled PET and chitosan were labeled rPET@CS, followed by the corresponding deposition rate (i.e., 10 mL/h, 7.5 mL/h, 5 mL/h and 2.5 mL/h).

For the supplementary layer, a 2% chitosan solution was prepared by dissolving 2 g of chitosan in a solution of 89 mL water and 9 mL of 1 N acetic acid under magnetic stirring. The prepared PET samples were immersed in this chitosan solution and left for 10 min under continuous magnetic stirring to ensure thorough coating. Subsequently, the samples were removed from the chitosan solution, briefly immersed in distilled water for 10 s to remove any excess chitosan and then dried in an oven for 7 h at a temperature of 40 °C.

### 2.3. Characterization Methods

The morphology of the recycled PET-based membranes was examined using a scanning electron microscope from FEI Company (Hillsboro, OR, USA). Images were captured by detecting the secondary electron beam at an accelerating voltage of 30 keV.

The integrity of the functional groups in the synthesized materials was evaluated using a ZnSe crystal FT-IR spectrometer (Nicolet 6700, Thermo Nicolet, Madison, WI, USA). Measurements were conducted at room temperature with 32 scans per sample, spanning a spectral range of 4000 to 600 cm^−1^ and a resolution of 4 cm^−1^. Data acquisition and analysis were carried out using Omnic software (version 8.2, Thermo Nicolet).

### 2.4. Antimicrobial Assay

The study utilized the following microbial strains: *Staphylococcus aureus* ATCC 25923, *Pseudomonas aeruginosa* ATCC 27853 and *Candida albicans* ATCC 10231. All strains were obtained from the strain collection of the Microbiology Laboratory, Faculty of Biology, University of Bucharest.

The capacity of the recycled PET samples to influence microbial adherence and biofilm production was evaluated using sterilized material fragments (1 cm × 1 cm). These fragments were placed in sterile 6-well plates, each well containing 2 mL of simple broth and 20 μL of microbial suspension (0.5 McFarland for bacteria or 1 McFarland for yeasts). After 24 h of incubation at 37 °C, the materials were washed with sterile physiological saline, and the medium was replaced to encourage biofilm development. The plates were incubated for 24, 48 and 72 h. After each incubation, the biofilm-coated samples were washed with sterile saline and transferred to tubes containing 1 mL of sterile saline. Vortexing (30 s) and sonication (10 s) detached the biofilm cells. Serial dilutions of the resulting cell suspensions were inoculated onto solid culture media to quantify colony-forming units (CFUs).

### 2.5. In Vivo Experiments

The in vivo experimental protocol adhered to the animal laboratory experiments guide of the Vasile Goldis Western University of Arad and received approval from the Ethical Committee. CD1 mice were maintained in IVC cages with controlled airflow, a 12 h light/dark cycle and constant temperature and humidity. Prior to the biological assay, all scaffolds were sterilized under UV light for 30 min per side.

The materials were implanted subcutaneously into a pocket in the dorsal region of the mice under anesthesia induced by intraperitoneal administration of xylazine/ketamine. The mice were randomly divided into five experimental groups (n = 20) as follows: control, rPET@CS 10 mL/h, rPET@CS 7.5 mL/h, rPET@CS 5 mL/h and rPET@CS 2.5 mL/h. In each group, 10 animals were euthanized after 24 h, and the remaining 10 were euthanized 7 days post-surgery.

Post-surgery, the animals were housed individually and monitored daily by a veterinary professional. Clinical evaluations included the assessment of surgical incision aspects, redness, infection, edema, abscess, hematoma and scarring. Biopsies were collected at 24 h and 7 days post-implantation under anesthesia, and blood samples were drawn via cardiac puncture for biochemical analysis.

#### 2.5.1. Biochemistry

The collected blood samples were centrifuged at 3500 rpm for 10 min to separate the serum. The serum was then analyzed for C-reactive protein (CRP) levels using a Mindray BS-120 chemistry analyzer (Shenzhen Mindray Bio-Medical Electronics Co., Ltd., Shenzhen, China) and the CRP FL reagent kit (Chema Diagnostica, Monsano, Italy).

#### 2.5.2. Histology

The explanted materials, along with the surrounding tissue (skin and underlying connective tissue), were fixed in a 4% paraformaldehyde solution, embedded in paraffin, sectioned at 5 μm and stained using hematoxylin and eosin (H&E) and Masson-Goldner trichrome. The stained microscopic sections were analyzed using an Olympus BX43 microscope equipped with an Olympus XC30 digital camera (Tokyo, Japan) and CellSens software, version 3.2. Histological scoring was conducted based on the degree of inflammatory infiltration, fibroblast activity and neovascularization. Each parameter was graded on a scale from 0 (not present) to 4 (extensive), reflecting the severity of the tissue reaction.

#### 2.5.3. Immunohistochemistry

Immunohistochemistry was performed on 5 µm tissue sections that were deparaffinized and rehydrated using standard protocols. Immunostaining was visualized with a Novocastra Peroxidase/DAB kit (Leica Biosystems, Nussloch, Germany), following the manufacturer’s instructions. The primary antibody used was a polyclonal anti-TNF-α antibody at a dilution of 1:100 (Santa Cruz Biotechnology, Santa Cruz, CA, USA).

Negative controls were conducted by substituting the primary antibody with irrelevant immunoglobulins of matched isotypes under identical conditions. The stained slides were analyzed using an optical microscope to assess the presence and localization of TNF-α expression.

#### 2.5.4. Immunofluorescence

Deparaffinized and rehydrated sections underwent antigen unmasking using sodium citrate buffer (pH 6.0) followed by blocking with BSA for 1 h. The sections were then incubated with the primary antibody F4/80 (Abcam, Cambridge, UK, dilution 1:100). Alexa Fluor dye-conjugated secondary antibody (dilution 1:500) was subsequently applied, and nuclei were counterstained with DAPI. Fluorescence signals were visualized using a Leica TCS SP8 confocal microscope (Wetzlar, Germany), enabling detailed observation of the stained sections.

## 3. Results

**Figure 1:**
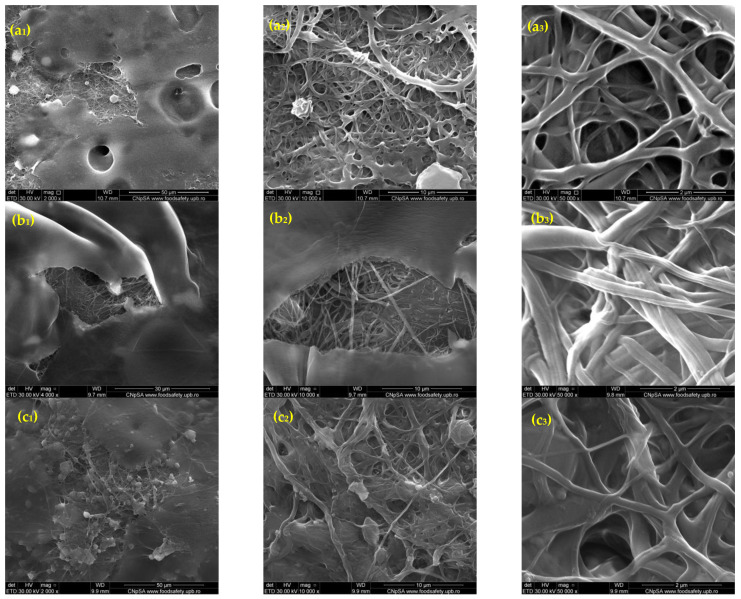

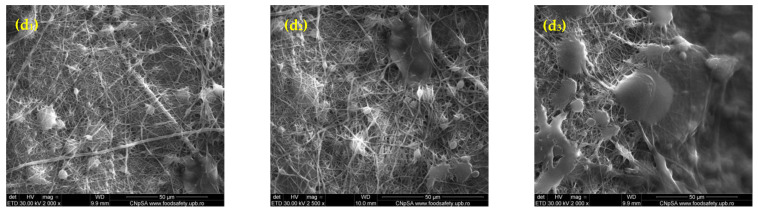
SEM images of rPET@CS samples obtained through electrospinning at different flow rates (10, 7.5, 5 and 2.5 mL/h)., where (**a1**–**a3**) rPET@CS/10, (**b1**–**b3**) rPET@CS/7.5, (**c1**–**c3**) rPET@CS/5 and (**d1**–**3**) rPET@CS/2.5.

The SEM images in Figure 1 illustrate the morphological characteristics of rPET@CS scaffolds fabricated through electrospinning at different flow rates (10, 7.5, 5 and 2.5 mL/h). The flow rate facilitates a more uniform deposition of fibers during electrospinning. At higher flow rates of 10 mL/h and 7.5 mL/h, the scaffolds exhibited a fibrillar structure embedded within a continuous chitosan layer. In contrast, the scaffolds prepared at lower flow rates of 5 mL/h and 2.5 mL/h demonstrated a more heterogeneous morphology, with both fibrillar structures and spherical formations. The spherical inclusions are a unique feature at these settings, potentially arising from instabilities during electrospinning, such as Rayleigh instability or phase separation. These spheres may form due to the slower flow rate reducing the stretching force on the polymer jet, resulting in droplets that solidify during deposition.

**Figure 2:**
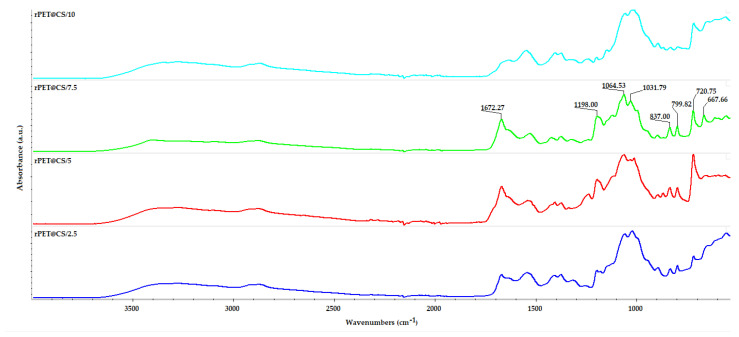
FT-IR spectra of PET@CS samples electrospun at different flow rates.

The IR spectra in Figure 2 represent the analysis of rPET@CS samples fabricated through electrospinning at different flow rates. The spectra confirm the presence of both rPET and CS, with characteristic bands observed across all samples. Key absorption bands include 3405 cm^−1^ (O–H and N–H stretching vibrations from chitosan), 2890 cm^−1^ (C–H stretching vibrations common to PET and chitosan), 1672 cm^−1^ (amide I band from chitosan) and 1198 cm^−1^ (C–O–C stretching from glycosidic linkages in chitosan). Additional bands related to PET were observed at 799 cm^−1^ and 724 cm^−1^ (aromatic C–H bending vibrations) and 667 cm^−1^ (C–H out-of-plane bending vibrations) [47,48,49].

**Figure 3:**
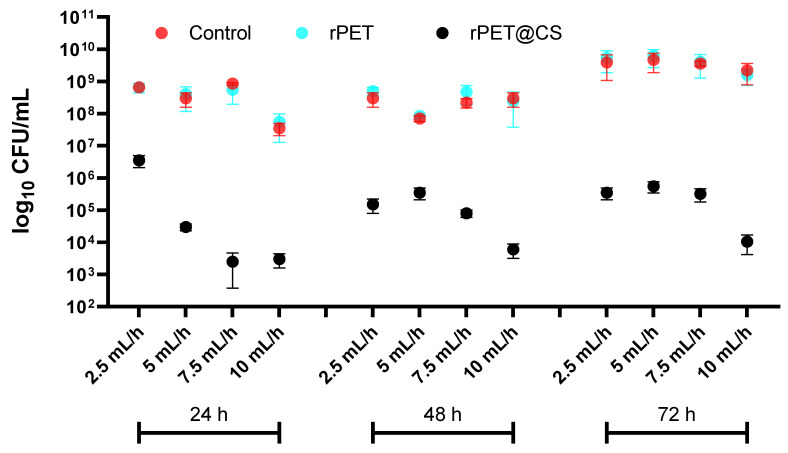
Logarithmic (log_10_ CFU/mL) quantification of *Staphylococcus aureus* adherence and biofilm formation on control, rPET and rPET@CS over 24, 48 and 72 h.

The study, visually summarized in Figure 3, evaluates the influence of electrospinning parameters on bacterial adherence and biofilm formation of *Staphylococcus aureus* on rPET fibers and rPET@CS compared to a control surface over 24, 48 and 72 h. The results indicate that bacterial colonization is significantly higher on both control and rPET surfaces, whereas the rPET@CS samples exhibit markedly lower bacterial adhesion and biofilm development. The observed reduction in bacterial load on rPET@CS suggests that chitosan coating provides antimicrobial properties, likely inhibiting initial bacterial attachment and delaying biofilm maturation. Additionally, variations in electrospinning parameters (2.5, 5, 7.5 and 10 mL/h) appear to influence bacterial colonization, with certain conditions favoring reduced adherence, potentially due to changes in fiber morphology, porosity or surface charge imparted during fabrication. Over time, biofilm formation becomes more established on control and rPET surfaces, while rPET@CS continues to show significantly lower bacterial loads, reinforcing its potential as an antimicrobial biomaterial.

**Figure 4:**
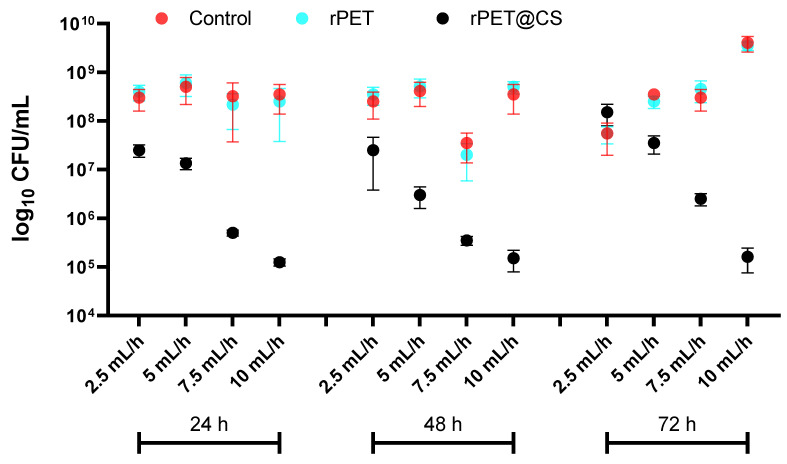
Logarithmic (log_10_ CFU/mL) quantification of *Pseudomonas aeruginosa* adherence and biofilm formation on control, rPET and rPET@CS over 24, 48 and 72 h.

The results in Figure 4 highlight the adherence and biofilm formation of *Pseudomonas aeruginosa* on different substrates (Control, rPET and rPET@CS) over 24, 48 and 72 h, under varying electrospinning parameters (2.5, 5, 7.5 and 10 mL/h).

Across all time points, both control and rPET surfaces exhibit high bacterial adhesion and biofilm formation, with CFU/mL values consistently in the range of 10^8^–10^10^, indicating that untreated rPET does not inhibit bacterial colonization. This suggests that the physicochemical properties of rPET remain favorable for *Ps. aeruginosa* attachment and biofilm maturation.

In contrast, rPET@CS shows a substantial reduction in bacterial adhesion, where CFU/mL values drop to 10^5^, indicating a strong inhibitory effect.

At 72 h, bacterial counts increase across all samples, reflecting biofilm progression. However, rPET@CS maintains significantly lower CFU/mL values, demonstrating sustained antimicrobial efficacy.

**Figure 5:**
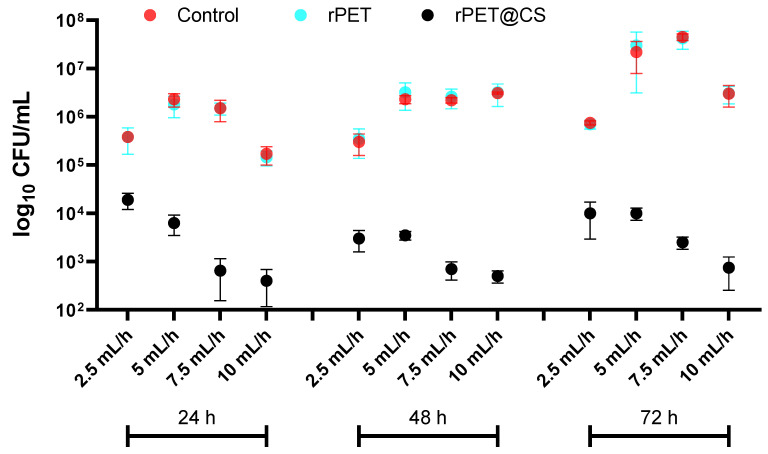
Logarithmic (log_10_ CFU/mL) quantification of *Candida albicans* adherence and biofilm formation on control, rPET and rPET@CS over 24, 48 and 72 h.

Figure 5 shows the results of the adherence and biofilm formation of *Candida albicans* on different substrates (over 24, 48 and 72 h) under varying electrospinning parameters. The fungal load, expressed as log_10_ CFU/mL, provides insights into *C. albicans* colonization behavior on these surfaces. Across all time points, both control and rPET surfaces exhibit high fungal adhesion and biofilm formation, with CFU/mL values consistently in the range of 10^5^–10^7^, indicating that untreated rPET does not inhibit *C. albicans* colonization. In contrast, rPET@CS shows a substantial reduction in fungal adhesion; CFU/mL values drop to 10^3^, demonstrating a strong antifungal effect. This inhibition is likely due to chitosan’s ability to disrupt fungal cell walls, interfere with adhesion mechanisms and impair biofilm matrix formation. At 72 h, fungal colonization increases across all substrates, reflecting biofilm progression. However, rPET@CS continues to maintain significantly lower CFU/mL values, reinforcing its sustained antifungal efficacy. The variations observed across different electrospinning parameters suggest that fiber morphology, surface roughness and porosity influence fungal adherence, with certain conditions enhancing chitosan’s antifungal properties.

The adhesion and biofilm formation of *Staphylococcus aureus*, *Pseudomonas aeruginosa* and *Candida albicans* on rPET@CS samples exhibit notable differences due to their distinct surface interactions and biofilm development strategies. *S. aureus* primarily adheres to surfaces through hydrophobic interactions and adhesins, forming biofilms composed of extracellular polymeric substances that provide structural integrity and resistance. Chitosan disrupts these initial adhesion processes by altering surface charge and interfering with biofilm matrix formation [50,51]. *Ps. aeruginosa* is a strong biofilm producer, relying on flagella- and pili-mediated adhesion, followed by EPS secretion regulated by quorum sensing. Its biofilms are particularly resilient, but chitosan can impair early-stage adhesion and communication, limiting maturation [50,52]. *C. albicans* follows a unique biofilm formation strategy, transitioning from yeast to hyphal growth, which enhances surface adherence and biofilm robustness. Chitosan inhibits this transition, reducing adhesion and weakening biofilm structure [53]. Overall, rPET@CS effectively hinders microbial adhesion and biofilm development, though the extent of inhibition varies among these strains due to their differing attachment mechanisms and biofilm architectures.

**Figure 6:**
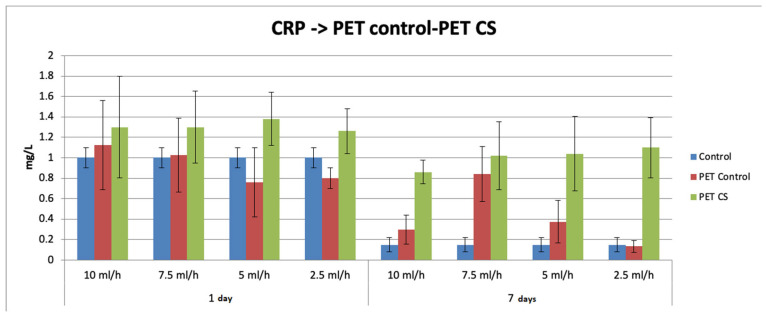
The effects of rPET@CS subcutaneous implantation in mice on the C-reactive protein (CRP) levels at 24 h and 7 days post-surgery.

Figure 6 shows the effects of the subcutaneous implantation of rPET@CS on the serum level of the inflammatory marker CRP. At 72 h after implantation, serum CRP concentration increased for all experimental groups, followed by a 14-day decrease. For both time intervals, the CRP level was significantly increased for rPET@CS implants compared to both the control and PET control.

**Figure 7:**
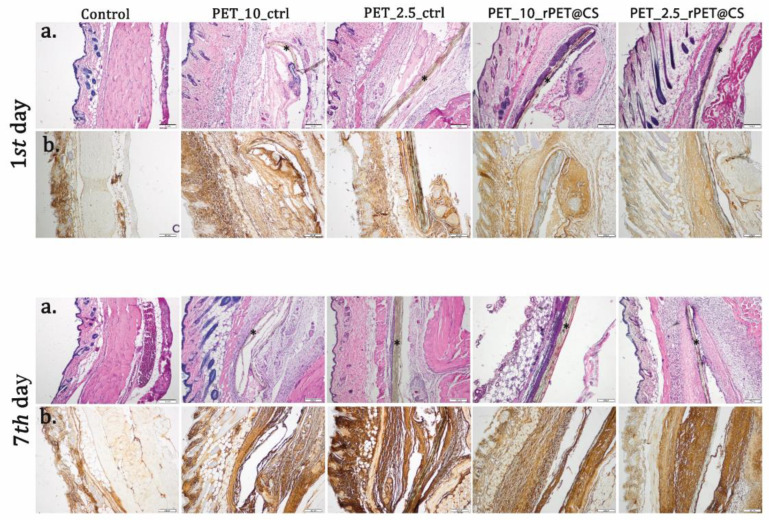
Biocompatibility analysis of rPET@CS at 24 h and 7 days post-implantation. (**a**) H&E stain; (**b**) TNF-α immunohistochemistry. Material (*); Barr 200 μm.

**Figure 8:**
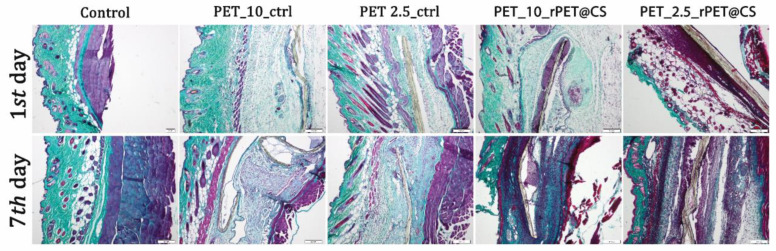
Collagen proliferation analysis after rPET@CS subcutaneous implantation at 24 h and 7 days by Masson-Goldner trichrome stain. Barr 200 μm.

Daily post-implantation clinical analysis revealed no local or systemic side effects (Figure 7 and Figure 8). Peri-implant edema was observed for PET control samples at 24 h, which was maintained at 7 days after surgery (Table 1). This reaction increased with the rate of fiber deposition. At 24 h after implantation, the presence of an inflammatory infiltrate in cutis and subcutis consisting mainly of polymorphonuclear neutrophils (PMNs) was observed. Otherwise, for rPET@CS, inflammatory cells are mainly localized near material.

After 7 days, there was a proliferation of collagen that promoted the formation of a fibrous capsule around the materials (Figure 8). CS-coated PET materials were surrounded by a clearly delimited area of granulation tissue consisting of fibroblasts, macrophages and proliferating capillaries.

Immunohistochemistry was performed for tissue sections to analyze inflammatory response towards the implanted materials. As shown in Figure 7, immunopositivity for TNF-α increased on connective tissue surrounding PET materials in a time-dependent manner. Immunoreaction was strong enough even though PET was coated with CS.

**Figure 9:**
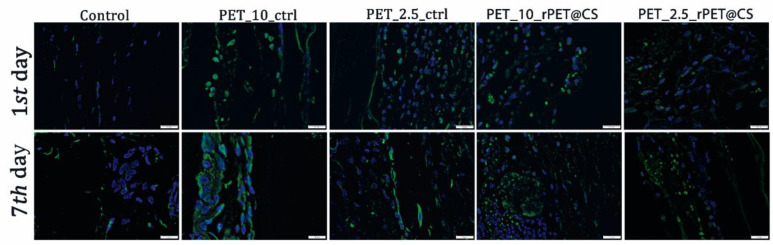
F4/80 protein expression as revealed by confocal microscopy at 24 h and 7 days post-implantation. F4/80 is labeled in green, and the nuclei are counterstained with DAPI.

The F4/80 marker was highly expressed in samples that had PET materials implanted, which highlights the activation of macrophages with increasing contact time with the material. Chitosan-coated PET showed a lower immunopositivity than non-coated materials (Figure 9).

## 4. Discussion

As both PET and CS have been demonstrated to exhibit advantageous properties for various applications, they have also been considered together in synergistic composites. Several research studies have reported different grafting methods to covalently link these two polymers and further use them as raw materials for films or non-woven textiles. The literature-reported grafted composites demonstrated potential applicability for food packaging and antimicrobial biomedical devices [35,43,44].

Differently, this study proposes the utilization of CS as a coating on recycled PET fibers. The electrospinning method was chosen to obtain these composite nanofibers due to a series of advantages linked to this technique [55,56,57,58]. Moreover, electrospun fibers exhibit properties of interest for further advanced applications, including high specific surface area, lightweight nature, excellent mechanical characteristics, high aspect ratio and tunable porosity [57,58].

Hence, considering the cost-effectiveness, simplicity and desirable outcomes of electrospinning, we utilized this method to obtain recycled PET–chitosan composite nanofibers suitable for biomedical applications. The electrospun fibers were further characterized through a series of physicochemical and biological analyses that demonstrated promising results and supported their future use for proposed applications.

The use of post-electrospinning chitosan coating was chosen for its simplicity and accessibility, avoiding the need for co-axial electrospinning setups, which require more complex control of solution parameters and dual-jet configurations [59,60]. While co-axial methods can provide deeper chitosan incorporation and more integrated release systems [61], the surface coating approach used in this study was adequate for achieving antimicrobial functionality in the short term and under physiological conditions, as demonstrated in our biological evaluations.

Numerous electrospun nanofibers have been recently reported as promising for different biomedical applications, including sustained drug release [62,63,64,65], dressings for enhanced wound healing [66,67,68], tissue engineering [69], biosensors [70], adjuvants in periodontal therapy [71] and antimicrobial membranes, textiles and coatings [4,68,72]. Similar to previously reported nanofibers, the materials developed in this study represent valuable candidates for future applications in biomedicine.

The antimicrobial efficacy and biofilm inhibition properties of chitosan-based materials have been widely explored, particularly in biomedical applications. Previous studies have shown that chitosan coatings can significantly reduce bacterial adhesion by altering surface charge, preventing biofilm maturation and disrupting microbial membranes [50]. Our findings align with these observations, as rPET@CS scaffolds demonstrated lower bacterial and fungal adhesion compared to untreated rPET and control surfaces.

Electrospinning parameters play an important role in shaping fiber morphology, which directly affects microbial interactions. Studies have reported that fiber diameter, porosity and surface roughness influence bacterial attachment, with smoother and more homogeneous fibers generally exhibiting reduced biofilm formation [52]. In this study, rPET@CS scaffolds fabricated at higher flow rates (10 and 7.5 mL/h) displayed a more fibrillar structure, while lower flow rates (5 and 2.5 mL/h) resulted in heterogeneous morphologies with spherical inclusions. Similar findings have been reported in electrospun nanofibrous membranes, where fiber morphology influenced microbial adhesion by modulating surface energy and hydrophobicity [51].

The chitosan used in this study had a medium molecular weight (MW) (~190–310 kDa) and a degree of deacetylation (DDA) (75–85%), which were chosen based on a balance between solubility, film-forming ability and antimicrobial activity. Medium-MW chitosan offers favorable viscosity and coating properties, while a moderate-to-high DDA ensures the cationic character necessary for disrupting microbial membranes [73,74]. Studies have shown that altering these parameters can influence antimicrobial efficacy, degradation rate and interaction with host tissue [75]. For example, lower-MW chitosan degrades faster and may be more suitable for applications requiring transient activity, whereas higher DDA enhances electrostatic interactions with bacterial cell walls [76,77,78]. These aspects may be tuned in future studies to match specific clinical needs.

Chitosan’s antimicrobial efficacy is attributed to multiple mechanisms, including electrostatic interactions with negatively charged microbial cell walls, interference with quorum sensing and inhibition of biofilm matrix formation. In Gram-positive *S. aureus*, chitosan disrupts peptidoglycan integrity, leading to increased membrane permeability and cellular leakage [79,80]. For Gram-negative *Ps. aeruginosa*, the presence of an outer membrane provides an additional defense mechanism, yet studies have shown that chitosan can still impair its adhesion and biofilm formation by disrupting flagella- and pili-mediated attachment [50,81]. In *C. albicans*, chitosan is known to inhibit the yeast-to-hyphal transition, a key process in fungal biofilm development, reducing adhesion and biofilm matrix synthesis [51,82]. These findings support our observation that rPET@CS scaffolds significantly inhibited microbial colonization compared to untreated rPET.

The in vivo testing in this study was limited to a 7-day period, which was selected to assess early-stage tissue response and acute inflammation. While this timeframe is sufficient to detect short-term compatibility and immediate immune responses, we acknowledge that longer-term evaluations—such as 21 or 28 days—would be beneficial to study scaffold integration, chronic inflammation and degradation. Future work will address these aspects through extended implantation studies.

Chitosan-coated biomaterials have been widely investigated in implantable devices to reduce microbial infections and modulate inflammatory responses. A recent review highlighted that chitosan-functionalized surfaces can promote tissue integration while minimizing bacterial biofilm formation, making them suitable for wound dressings, catheters and orthopedic implants [52]. The inflammatory response to rPET@CS implantation observed in this study is consistent with prior reports, where transient increases in C-reactive protein (CRP) levels and macrophage recruitment were observed following chitosan-coated biomaterial implantation [51,83,84]. However, chitosan-modified surfaces generally elicit a lower inflammatory response than uncoated materials, which is supported by our immunohistochemical analysis showing reduced TNF-α expression and macrophage activation near rPET@CS implants.

Although the chitosan coating was effective within the study window, we recognize that its long-term stability under physiological conditions remains to be fully characterized. Future studies will include degradation and release kinetics of chitosan coatings to better understand their persistence and sustained performance in vivo.

Given the broad-spectrum antimicrobial properties and favorable tissue response of rPET@CS scaffolds, these materials hold potential for applications in medical textiles, wound healing and implant coatings. Further studies should explore the long-term stability of chitosan coatings, as well as their performance in in vivo infection models. Additionally, optimizing electrospinning parameters could enhance the antimicrobial efficacy of these materials by fine-tuning fiber morphology and surface properties. Future research should also investigate the synergy between chitosan and other bioactive agents, such as metallic nanoparticles, to further improve antimicrobial resistance.

## 5. Conclusions

This study highlights that rPET@CS scaffolds, fabricated via electrospinning, exhibit strong antimicrobial and antibiofilm properties against *S. aureus*, *Ps. aeruginosa* and *C. albicans*. The chitosan coating effectively reduced microbial adhesion and biofilm formation, highlighting its potential as an antimicrobial agent. Additionally, implantation studies revealed a transient inflammatory response, with macrophage recruitment and collagen deposition indicating favorable tissue integration. Given their antimicrobial efficacy and biocompatibility, rPET@CS scaffolds hold promise for biomedical applications such as wound dressings, implant coatings and infection control. Future research should optimize electrospinning parameters, assess long-term stability and explore synergistic bioactive enhancements. These findings support the use of sustainable, chitosan-coated PET scaffolds in advanced biomaterial applications.

## Figures and Tables

**Table 1:** Tissue reactions by histometric scoring used to grade inflammation and neovascularization in the tissue surrounding subcutaneous implants.

Material	Implantation Period (Days)	Edema	PMNs	M	F	NV
Control	1	-	+	-	-	-
	7	-	-	+	-	-
PET 10 mL/h	1	++++	+++	++	+	-
	7	+++	++	+++	++++	-
PET 7.5 mL/h	1	+++	+++	+	+	-
	7	++	+	+++	+++	-
PET 5 mL/h	1	++	+++	+	+	-
	7	++	+	+++	++	+
PET 2.5 mL/h	1	++	+++	+	+	-
	7	+	+	+++	++	+
rPET@CS 10 mL/h	1	++	++++	++	+	-
	7	+	+	++++	++++	++
rPET@CS 7.5 mL/h	1	+	+++	+	+	-
	7	+	+	++++	++++	++
rPET@CS 5 mL/h	1	+	+++	+	+	-
	7	-	+	+++	+++	++
rPET@CS 2.5 mL/h	1	+	+++	+	+	-
	7	-	+	+++	+++	++

PMNs: polymorphonuclear neutrophils; M: macrophages; F: fibroblasts, NV: neovascularization. Tissue reactions are rated from - (not present) until ++++ (extensive). Control samples are also available in [46,54].

## Data Availability

Data can be obtained from the authors by request.
